# Immediate Implants in the Aesthetic Zone: Is Socket Shield Technique a Predictable Treatment Option? A Narrative Review

**DOI:** 10.3390/jcm10214963

**Published:** 2021-10-26

**Authors:** Nicola De Angelis, Antonio Signore, Arwa Alsayed, Wong Hai Hock, Luca Solimei, Fabrizio Barberis, Andrea Amaroli

**Affiliations:** 1 Department of Surgical and Diagnostic Sciences, University of Genoa, 16132 Genoa, Italy; dr.signore@icloud.com (A.S.); difp_as@yahoo.com (A.A.); whhock@yahoo.com (W.H.H.); lucasolimei@hotmail.it (L.S.); 2 Department of Dentistry, University Tunku Abdul Raman (UTAR), Sungai Buloh 47000, Malaysia; 3 Therapeutic Dentistry Department, Faculty of Dentistry, First Moscow State Medical University (Sechenov University), 119991 Moscow, Russia; 4 Department of Civil, Chemical and Environmental Engineering, University of Genoa, 16100 Genoa, Italy; fabrizio.barberis@unige.it; 5 Department of Orthopedic Dentistry, Faculty of Dentistry, First Moscow State Medical University (Sechenov University), 119991 Moscow, Russia

**Keywords:** dental implant, socket-shield technique, bone resorption, immediate implant

## Abstract

(1) Background. Dental implant placement in the anterior region requires extreme precision due to relatively high aesthetic demand. This narrative review aimed to analyse some of the available clinical studies of the socket-shield technique and determine its viability for dental implant survival/success and complication rates. (2) Methods. An electronic search for publications was performed using the Cochrane, PubMed-MEDLINE, Web of Science, and Google Scholar databases. All electronic searches included human clinical and animal studies and were performed by three independent examiners. (3) Results. A total of 1383 records were identified with the initial search strategies, but only 25 full texts + five abstracts clinical studies were kept after the recruitment criteria screening. The technical details, advantages, and limitations of the techniques were illustrated. (4) Conclusion. Within the limitations of the present review, it would be merely justified that immediate dental implant placement in conjunction with the socket-shield technique can be a promising strategy for dental implant therapy.

## 1. Introduction

Tooth loss and the resulting trauma to the hard tissue are accompanied by multiple dimensional changes and remodelling of the alveolar ridge [1,2,3].

Araujo and Lindhe [3] showed that the alveolar ridge buccal dimension experiences horizontal resorption of about 56% and a reduction of the lingual/palatal bone wall of 30% during the four month interval following tooth extraction. Similarly, Schropp et al. [2] observed overall alveolar ridge bone reduction in the horizontal dimension following a tooth extraction, and the resorption of the buccal part of the alveolar ridge is more evident than the lingual part.

In this way, the morphology of the healed alveolar ridge after the tooth extraction almost shows up as a discrepancy of ±2 mm in bone height between the lingual and buccal bone plates of the alveolar ridge [4]. The pattern of the healing includes a biological change of the socket volume, due to the resorption of the most coronal part of the alveolar bone (bundle bone) which is connected to the periodontal fibers of the tooth. Araujo and Lindhe [4] in an animal study histologically demonstrated that marked dimensional alterations occurred during the first eight weeks following the extraction of mandibular premolars. In this interval, there was a consistent osteoclastic activity resulting in resorption of the crestal region of both the buccal and the lingual bone wall. The reduction of the height of the walls was more pronounced at the buccal than at the lingual aspect of the extraction socket. The height reduction was accompanied by a “horizontal” bone loss that was caused by osteoclasts present in lacunae on the surface of both the buccal and the lingual bone wall.

Unfortunately, this condition can interfere with the optimal placement of dental implants and frustrate the overall aesthetic outcomes of prostheses.

In order to overcome the side effects, approaches such as immediate dental implant placement [5], the use of both graft materials [6] and barrier membranes [7], and complementary approaches by light therapy [8], the implant placement in a more palatal position and/or immediate temporary crown [9] have been investigated and studied, to preserve the alveolar ridge bone dimension. The challenge of the healing after the extraction has been described also in terms of residual thickness of the buccal bone, especially in aesthetic zones [10,11,12,13]. Braut et al. [10] in a retrospective study conducted on 125 cone beam computed tomographies, and 498 anterior teeth concluded that the facial bone wall in the crestal area was either missing or thin in roughly 90.0% of patients

Misawa et al. [13] investigated the dimensional alterations that occurred in the alveolar process of the incisor and premolar sites of the maxilla following tooth removal through a CBCT analysis. Results showed that the overall cross-sectional area was reduced from 99.1 to 65.0 mm, the height from 11.5 to 9.5 mm, and the width from 8.5 to 3.2 mm, 8.9 to 4.8 mm (middle portion), and 9.0 to 5.7 mm (apical portion).

The regenerative procedure as it attempts to reduce these dimensional changes, such as bone substitutes in the alveolar sockets or external to the buccal bone plate, may reduce the resorption, but showed contradictory evidence and not always satisfactory preservation of the dimensions of the alveolar ridge bone [14].

**Figure 1:**
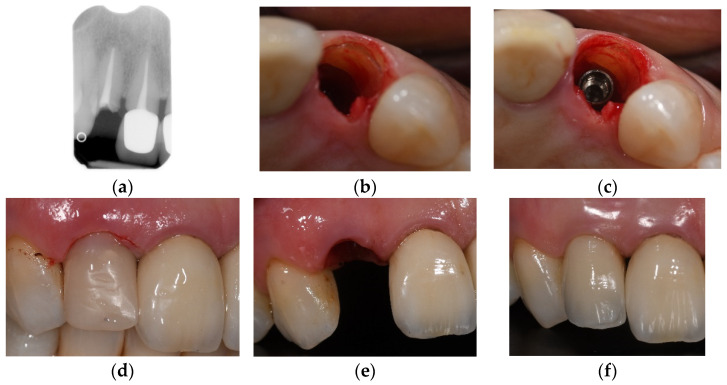
Case of socket shield technique and immediate implant placement. (**a**) the preoperative X-ray shows the absence of apical and/or periodontal infection (**b**,**c**) the root shield is preserved buccally and slightly more apical from the gingival margin (**d**) temporary crown in acrylic resin- no functional loading (**e**) emergence profile 3 months after the procedure (**f**) one year follow up.

Therefore, the predictability of the appearances of the hard and soft tissues after reconstructive surgical interventions is limited because horizontal and vertical bone augmentations are frequently accompanied by subsequent tissue shrinkage [11]. Speculation surrounding tooth root retention in the alveolar ridge and its possible influence on the prevention of the bone resorption process have been made [15,16,17,18]. Basically, clinical studies demonstrated retaining decoronated tooth roots in the alveolar process may preserve the alveolar ridge bone dimensions at the extraction site and enable vertical bone growth, which can be observed coronally to the decoronated tooth root [19,20,21]. A case of socket shield is displayed in Figure 1.

In order to meet the demand for more predictability of the post-surgical gingival conditions, Hürzeler and collaborators [21] described the effectiveness of immediate dental implant placement in conjunction with the socket-shield technique for buccal alveolar ridge bone preservation after tooth removal in the anterior region.

In our review, with intervention as the main objective, immediate dental implant placement in conjunction with the socket-shield technique was considered with respect to the classical approach (extraction and immediate implants with bone grafts); as well as complications such as survival and success rates. As secondary objectives, buccal bone preservation/remodelling and final outcomes were also included.

## 2. Materials and Methods

All electronic searches included human clinical studies and were restricted to the last ten years with the following databases: PubMed-MEDLINE, Web of Science, and Google Scholar. Article researches were performed using the following terms: *socket-shield technique, immediate implant, and bone resorption.*

Arbitrary inclusion criteria for the articles in this review, included immediate dental implant placement using the socket-shield technique, and immediate dental implant placement with bone grafts in the aesthetic zone. Human clinical randomized control trials, case-control studies, case series, case reports, and some explicative animal studies available in the English language and on journals with the peer-review process before publication were considered. Exclusion criteria were articles not related to the topic and not written in English.

## 3. Results

A total of 1383 records were identified with the initial search strategies, but only 25 full texts + five abstracts clinical studies were kept after the recruitment criteria screening. The details of the included clinical studies on the immediate dental implant placement in conjunction with the socket-shield technique are shown in Table 1.

The outcomes in the clinical studies in the present review were assessed through clinical and radiological results. Dental implant failures were defined as detrimental effects until implant extraction, while clinical outcomes were defined as complications and adverse effects, such as dental implant exposures, tooth root fragment exposures, tooth root fragment infections, tooth root fragments with deep probing pocket depth, and tooth-root fragment migration. Simultaneously, the radiological outcomes consisted of buccal/proximal/crestal bone loss, including the above-mentioned clinical situations.

Three-dimensional bone loss around dental implants has been considered as an inevitable factor, following the tooth extraction and data are available in Table 2.

A total of 25 included clinical studies were enrolled in the present review, including nine case reports, eight case series, three randomized controlled clinical trials, two therapy protocols, one case-cohort study, one technical report, one case-control study.

**Figure 2:**
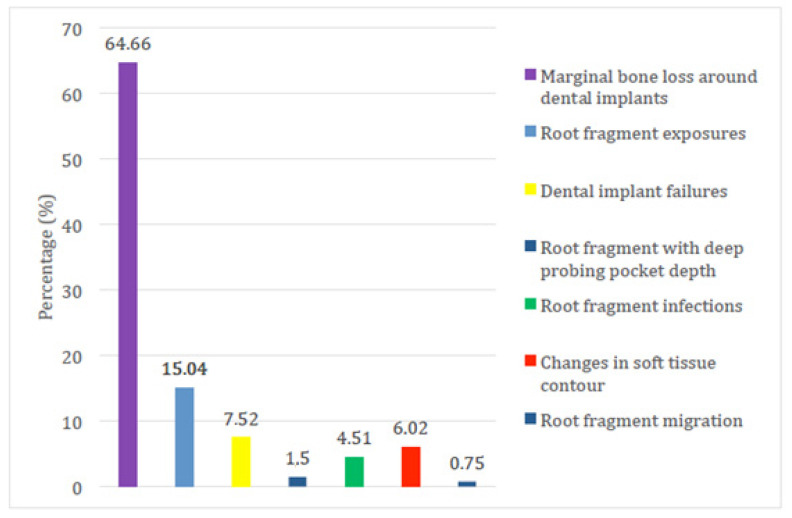
The frequency and percentage distribution of the dental implant failures and complications and undesired adverse effects of immediate dental implant placement in conjunction with the socket-shield technique of the included clinical studies. Bone loss as mentioned in the result section has been reported but considered inevitable.

A total of 537 subjects were included in the analyzed studies, with 570 immediate dental implant placements in conjunction with the socket-shied technique. Ten im-plants failed and were removed (1.75%), 123 implants (21.58%) showed different complications (such as marginal bone loss around dental implants, root fragment exposures, root fragment infections, root fragment with deep probing pocket depth, changes in soft tissue contour and root fragment migrated) as reported in Figure 2 and Table 3 and Table 4.

The cumulative survival rate for the 570 dental implants placed immediately in conjunction with the socket-shield technique was 98.25%, in spite of the fact that some complications had been treated successfully. Dental implant failures were due to an initial lack of osseointegration (seven out of 10 cases) or later peri-implantitis (three out of 10 cases).

## 4. Discussion

Clinical studies have suggested that retaining roots of hopeless teeth in conjunction with the immediate placement of the implant (socket shield technique) may avoid tissue alterations after tooth extraction and, to corroborate these evidences, several animal histologic studies have been addressed to the topic. Baumer, Hurzeler et al. (2013) [26] observed that new bone was visible between the implant surface and shield, as well as inside the vertical drill line. No osteoclastic remodelling of the coronal part of the buccal plate was observed. The clinical volumetric analysis showed a mean loss of 0.88 mm in the labial direction, with a maximum of 1.67 mm and a minimum of 0.15 mm.

Zhang et al. (2019) [47] compared 32 extraction sockets divided into four groups in a canine study. The first group (T1) was left with the bone clot, the second (T2) was filled up with a xenograft bovine bone substitute (Bioss Collagen Geistlich), in the third (T3), the socket shield was performed in conjunction with the natural blood clot, in the fourth T4) a xenograft bovine bone substitute (Bioss Collagen Geistlich) was added to the socket shield. Width, height, and dimensional alterations of T3 and T4 were significantly lower than those of T1 and T2. Bone morphological parameters displayed no significant differences among the four groups except for the trabecular thickness of T1 and T2. The quantity and quality of hard tissue containing the residual teeth of T3 and T4 were greater than those of T1 and T2.

Similar conclusions were addressed by Vina Almunja et al. [48] in a literature review published in 2013.

Schwimer et al. (2018) [39], in a case report, described the first histological results on humans and demonstrated that the bone entirely fills the space between root dentin and an osseointegrated implant surface.

### 4.1. Implant Failure and Marginal Bone Loss

Marginal bone loss and subsequent changes in the soft tissues can be described as an inevitable biological adjustment after tooth extraction. Mitzias MM. et al. [49] tried to analyze longitudinal volumetric changes during immediate implant placement with simultaneous intentional retention of the buccal aspect of the root. This study assessed 10 cases two years after periodontal ligament (PDL)-mediated immediate implant placement. Gypsum casts were scanned using a laser scanner and converted into digital three-dimensional rendered files. The digital casts were superimposed to observe the dimensional changes that occurred to the soft tissues. All changes were non-inferior to pre-extraction baseline measurements based on a 0.5-mm non-inferiority margin.

Thus, they concluded that the intentional retention of the buccal aspect of the root, with its periodontal apparatus during immediate implant placement, led to optimal soft tissue dimensional stability in the esthetic zone.

Eight-six cases of marginal bone loss around the dental implants within the mean range 0.15–1.3 mm were found in studies by Baumer et al. (2013), Abadzhiev et al. (2014), Baumer et al. (2017), Bramanti et al. (2018), Chen and Chen (2016), Chen and Pan (2013), Troiano et al. (2014), and Zhu et al. (2018) [22,26,27,28,29,31,43,45]. The reason for crestal bone loss in the study by Troiano et al. was the tooth root being worn down into a concave shape and positioned apically 2–3 mm from the bone crest where a modification of the socket-shield technique named the Root-T-Belt method was used. Abadzhiev et al. showed a combination of the socket-shield technique with bone graft and the exposure of the implant surface was attributed to an early infection of the bone substitute.

In the study by Baumer et al. consecutive patients treated with 10 immediate dental implant placements in conjunction with the socket-shield technique were evaluated for five years. Mean loss of marginal bone level at the dental implant shoulder amounted to 0.33 ± 0.43 mm at the mesial and 0.17 ± 0.36 mm at the distal aspects of the dental implants. The authors justified that immediate dental implant placement in conjunction with the socket-shield technique would be able to reduce the invasiveness of the surgical procedure, offering high aesthetic outcomes with effective preservation of facial tissue contours.

Bramanti et al. evaluated the marginal bone level at three years of follow-up both in immediate implants with and without socket-shield preservation. A lower rate of crestal bone resorption was recorded in the 20 socket-shield technique test group (0.605 ± 0.06 mm), while in the control group, the rate of crestal bone resorption was 1.115 ± 0.131 mm. The authors concluded that the socket-shield technique is a safe surgical technique that allows for dental implant rehabilitation characterized by better aesthetic outcomes. One of the possible reasons for this manifested stability of the therapy over time might be attributed to the maintenance of the vascular support provided by the periodontal ligament maintained to the tooth root portion left in situ. Despite marginal bone loss around dental implants being observed in 59.46% of cases (66 out of 111 cases), the range of 0.17–1.3 mm can be considered acceptable for long-term maintenance.

There are concerns that the socket-shield technique can only be used in immediate dental implant placement when the buccal tooth root fragment is intact, but in many cases, the tooth that has to be replaced is vertically fractured. In the study by Baumer et al. (2013), they evaluated if the socket-shield technique also works when the buccal tooth root fragment shows a vertical fracture line. In the study, the effect of separating the buccal tooth root fragment in the extraction socket into two pieces before immediate dental implant placement was assessed. It was found that there was no remodelling in the most coronal part of the crestal bone at the buccal site. The new bone formation could be observed between the dental implant and the tooth root fragments, as well as in the gap created between the two remaining tooth root fragments.

The reported reasons for dental implant failures in the study by Siormpas et al. (2014) and 2018 [40,41,42,43,44,45,46,47,48,49,50,51] were tooth root fragment infections causing peri-implantitis (three out of five cases) and dental implant mobility in the absence of infection, which failed to osseointegrate (two out of five cases). In this study, it was demonstrated that the socket-shield technique is able to present a better long-term success rate comparable to those of conventional immediate dental implants. In the study by Gluckman et al. (2017) [33], the reason for the five dental implant failures was the failure of osseointegration, but it was demonstrated that the socket-shield technique is able to perform competitively compared to the dental implant survival rates in both conventional immediate and delayed dental implant placement.

### 4.2. Root Fragment Exposure

Tooth root fragment exposures in terms of the coronal portion of the tooth root fragment perforating the soft tissue may be denoted as internal (toward the restoration) or external (toward the oral cavity) exposures of the tooth root fragment. In this review, tooth root fragment exposures were mostly reported in the study by Gluckman et al. (2017) [33] (16 out of 128 cases), followed by Cherel and Etienne (2014) [30] (two out of 19 cases), and Abitbol et al. (2016) [24] (one out of 19 cases). In the study by Gluckman et al. (2017), there were 12 internal and four external tooth root fragment exposures. All 12 internal exposures were managed by either no treatment and observation or by reduction of the exposed tooth root portion with a diamond bur coupled to a high-speed handpiece, while the four external exposures were managed by reducing the coronal aspect of the soft tissue closure. The authors justified that tooth root fragment internal exposures might be due a to lack of adequate space between the coronal edge of the tooth root fragment and the subgingival contour of the crown. In addition, the authors also clarified that external exposures can be related to an overextension of the coronal aspect of the tooth root fragment or the sharp coronal aspect that perforates the overlying soft tissues. In order to overcome the incidence of tooth root fragment exposures, the authors recommended reducing the tooth root fragment to the alveolar ridge bone crestal level and creating a chamfer in the crestal 2 mm of the tooth root fragment.

### 4.3. Bone Grafts and the Socket-Shield Technique

In the study by Botticelli et al. (2003) [50], the authors concluded that, if the distance between the dental implant surface and the socket wall is 0.5–1.0 mm, there is no need for a bone graft to fill the space. On the other hand, if the gap between the surface of the dental implant and socket wall is more than 1 mm, a grafting procedure is indicated. Out of the 19 clinical studies in the present review, seven clinical studies demonstrated grafting procedures in the gaps between the implant surfaces and the socket walls with immediate implant placement in conjunction with the socket-shield technique. In the study by Dayakar et al. (2018) [32], the space between the surface of the dental implant and the socket wall was filled with the bone graft, as the lingual jumping distance was more than 1 mm, and its clinical outcome had presented with no complications. In the study by Cherel and Etienne (2014) [30], a non-resorbable bone substitute of the deproteinized bovine bone mineral was placed to fill the gap between the dental implant and buccal plate. In the study by Abitbol et al. (2013) [24], in all 23 dental implants placed immediately in conjunction with the socket-shield technique, the bone substitute particles were integrated without signs of inflammation. In addition, in the study by Petsch et al. (2017) [38], the gap between the remaining lateral and lingual bone walls and the body of the dental implant was filled with xenograft bone material. After 24 months of follow-up duration, neither a clinical change of soft tissue was indicated nor retaining of tooth root fragments was presented. The same results were also presented by other articles included in this review.

By means of the presented results, it can be concluded that all the immediate dental implant placements in conjunction with the socket-shield technique, which were grafted with bone substitutes, demonstrated only tooth root fragment exposures in the studies by Cherel and Etienne (two out of two implants) and Abitbol et al. (one out of 23 implants), but no infection-related complications were observed.

Markus B. Hürzeler and his team described immediate dental implant placement in conjunction with the socket-shield technique [21] in a decoronated tooth approximately 1 mm apical to the gingival margin. One after the other, the osteotomy drills were performed through the lingual aspect of the tooth root, and all tooth root fragments were removed on the lingual, mesial, and distal aspects, retaining only the buccal portion of the tooth root fragment. Following continuous application of an enamel matrix derivative, the dental implant was inserted and positioned slightly apical to the retained tooth root fragment. The authors also justified that retaining the buccal aspect of the tooth root during dental implant placement does not appear to interfere with osseointegration without an inflammatory or resorptional response and may be beneficial in preserving the buccal bone plate. In the study by Han et al. [36] dental implants were immediately placed using the modified socket-shield technique with a 1.5 mm thick buccal portion of the tooth root fragment and with the most coronal portion at the bone crestal level. The root fragment was kept at least 1.5 mm to ensure resistance to fracture and resorption. The coronal part of the tooth root fragment was bevelled to make a lingual slope for a better emergence profile. All dental implants were placed slightly 2 mm apical to the crestal bone level, and no graft material was placed in the gap. After one year of follow-up, implant survival rates were recorded as 100%, and no biological complications were encountered. Aslan (2018) [25] introduced a modification of the socket-shield technique with the retention of a thin tooth root fragment. The buccal half of the tooth root was prepared until a crescent-shaped 1 mm thickness of the tooth root fragment was formed. The height of the tooth root fragment was reduced to 2 mm apical to the gingival margin and 1 mm coronal to the buccal bone crest for the purpose of biological width. The dental implant was then inserted and positioned 4 mm apical to the prospective gingival margin and away from the buccal tooth root fragment. The remaining gap between the tooth root fragment and surface of the dental implant was grafted with demineralized bovine bone material. Then, cone beam computed tomography confirmed the presence of a very thin buccal bone plate of 0.39 mm after 1 year of follow-up. Volumetric alteration analyses had demonstrated that remodelling of the alveolar bone following tooth extraction significantly influences the soft and hard tissue volumes. The buccal site showed 0.02 mm of change at the most coronal aspect. The author justified that an improvement in volume and contour stability can be obtained by retaining a thin tooth root fragment in immediate dental implant placement in conjunction with the socket-shield technique. Guo et al. [35] introduced a further modification of the socket-shield technique in combination with platelet-rich fibrin. The top of the retained tooth root was located parallel to the alveolar crest, and the tooth root fragment was prepared into a shape that was 1 mm thick. The dental implant was then placed at the lingual site of the retained tooth root fragment and two pieces of platelet-rich fibrin were placed in the gap in between the tooth root fragment and dental implant. The peri-implant tissue was well preserved, and no significant resorption of the peri-implant tissue was displayed after 18 months of follow-up.

In the present review, Cherel and Etienne applied immediate dental implant placement in conjunction with the socket-shield technique on two adjacent teeth. Instead of buccal tooth root fragment retention, the proximal tooth root fragments were left intact to preserve the papilla bone peak and immediate provisionalisation was carried out. Within 11 months of follow-up, the clinical and radiographic parameters for the inter-proximal papillae, gingival buccal margin levels, and inter-proximal bone levels showed no change. According to the authors, the modified socket-shield technique with intentionally retained proximal tooth root fragments allowed for the full three-dimensional preservation of alveolar architecture around two adjacent dental implants.

In the present review, almost all of the clinical case reports (seven out of eight studies) using immediate dental implant placement in conjunction with the socket-shield technique were documented for dental implant placement in the anterior aesthetic zone, while the impact of this technique has not been widely demonstrated in the posterior region of the alveolar ridge. The posterior alveolar buccal crest bone ridge may incur aesthetic compromise when substantial posterior alveolar ridge bone dimension loss is evident. In addition, the profound loss of posterior alveolar ridge bone dimension may result in vestibular depth reduction and lack of attached keratinized tissue [35]. In the study by Chen and Pan [31], a single immediate dental implant placement in conjunction with the socket-shield technique was documented in the posterior region of the right maxillary second premolar. Four months after dental implant placement, clinical examination showed healthy peri-implant soft tissue, and the alveolar ridge bone was well preserved.

Kan et al. (2013) [52] described the use of a proximal socket shield, which consists in leaving the proximal part of the root instead of the buccal one in order to preserve the interdental papilla, especially when there are implants on the adjacent sites.

Immediate post-extractive implants are currently widely used, but it is not clear whether they decrease the bone resorption that occurs after tooth extraction or not, nevertheless, several approaches for augmenting post-extractive sites are currently used and some of these have been evaluated in randomised controlled trials [53,54]. However, it is still unclear if these approaches can consistently reduce the horizontal and vertical resorption and which could be the best augmentation technique. [55].

In a randomised controlled clinical trial published in 2011 [14], the authors attempted a comparison between immediate implant placement with and without bone grafting. Seven out of 74 implants placed failed and this higher rate was attributed also to possible infections of the grafts.

In the studies by Cherel & Etienne and Abitbol [24,30] the immediate implant placement was conducted with socket shield preservation with bone substitute grafting in the marginal gap and no infections were reported by the authors.

Moreover, Markus B. Hürzeler [21] justified that retaining the buccal aspect of the tooth root during dental implant placement does not appear to interfere with osseointegration without an inflammatory or resorptional response.

From the clinical and scientific point of view, it seems that retaining the buccal portion of the roots increases the stability of the hard and soft tissues, without exposing the implants to infections.

In a previously published systematic review [56] the authors concluded that SST should not be used in routine clinical practise until a higher level of evidence is established and further RCT on SST is required to establish the clinical efficacy of this technique.

Based on the number of included studies (11 case reports, 6 case series, 1 human randomized control trial, 1 technical report, and 2 animal RCT), the conclusions are coherent, but it might be possible that too many restrictions were used for the quality assessment of the review, which excludes other two randomized clinical trials.

Therefore, the results could be strictly adherent to the inclusion and exclusion criteria, but, at the same time, might not provide enough clinical information about the real effectiveness of the technique.

## 5. Conclusions

Within the limitations of the present narrative review, and considering also the risk of bias in the selection of the articles, dental implant failures, and other complications and undesired adverse effects were evaluated in order to justify that the socket-shield technique can be a more promising strategy and therapy regimen for immediate dental implant placement due to the fact that preserving buccal tooth root fragment leads to the preservation of bundle bone and, therefore, the preservation of buccal bone volume. The comorbidities of surgical dental implant placement are reduced because additional surgical procedures and post-surgical pain and infections are avoided; it might offer a feasible treatment option to better fulfill the pink aesthetics demand. In order to confirm these preliminary conclusions, long-term follow-up of clinical studies should be conducted in order to substantiate the validity and to prove positively the extreme importance of preserving buccal tooth root fragments in the socket-shield technique for the sake of ascertaining the extremely high long-lasting, and satisfactory long-term clinical prognosis, as well as the plausibility of its therapy regimen outcomes.

Authors should discuss the results and how they can be interpreted from the perspective of previous studies and of the working hypotheses. The findings and their implications should be discussed in the broadest context possible. Future research directions may also be highlighted.

## Figures and Tables

**Table 1:** Clinical studies on immediate dental implant placement in conjunction with the socket-shield technique and its clinical therapy regimen outcomes. Abstracts are not included in the table.

No.	Authors and Publication Year	Type of Study	Sample Size (Number of Patients/Dental Implant)	Dental Implant placement Site/Loading Protocol	Duration n of Follow-Up (Months)	Grafting Materials	Clinical Therapy Regimen Outcomes
1	Abadzhiev et al., 2014 [22]	Prospective case- control study	25 patients/ 26 dental implants (10 SST; 16 conventional technique)	Alveolar ridge anterior region (SST)/not specified	24	Xeno- bone graft material	Mean crestal bone loss of 0.8 mm (SST *); 5 mm (control group)
2	Abd-Elrahman et al., 2020 [23]	Randomized clinical trial	25 patients/ 40 dental implants (20 SST; 20 control)	Alveolar ridge anterior region/ immediate	6	Graft not mentioned	One internal root fragment exposure, which did not require treatment, mean horizontal bone loss (0.15 mm SST; 0.32 mm control group), mean vertical bone loss (0.31 mm SST; 0.7 mm control group)
3	Abitbol et al., 2016 [24]	Case series, retrospective study	20 patients/23 dental implants	Not mentioned/immediate	12	Xenograft/allograft	Probing pocket of 8 mm in the mesio- buccal part of one root fragment; one root fragment exposure
4	Aslan 2018 [25]	Case report, prospective study	One patient/one dental implant	Alveolar ridge anterior region/immediate	12	Demineralized bovine bone	Thin buccal bone plate loss of 0.39 mm, natural convex buccal contour equal to adjacent central incisor
5	Baumer et al., 2013 [26]	Case report, pilot study	One patient/one dental implant	Alveolar ridge anterior region/delayed	6	No graft	Mean labial bone loss of 0.88 mm (range 1.67–0.15 mm)
6	Baumer et al., 2017 [27]	Case series,	10 patients/	Alveolar ridge	51–63	No graft	Marginal bone loss of 0.33 ± 0.43 mm
		retrospective pilot study	10 dental implants	Posterior region/four immediate six delayed			at mesial aspect and 0.17 ± 0.36 mm at distal aspect
7	Bramanti et al., 2018 [28]	Randomized controlled trial, prospective study	40 patients/40 dental implants (20 SST; 20 Conventional technique)	Alveolar ridge anterior region/immediate	36	Allograft (control group)	Marginal bone loss 0.605 ± 0.06 mm (SST); 1.115 ± 0.131 mm (control group)
8	Chen and Chen 2016 [29]	Case series, preliminary clinical study	four patients/four dental implants	Alveolar ridge anterior and posterior regions/not specified	3	Graft not mentioned	Mean buccal bone loss 0.83 ± 0.178 mm
9	Cherel and Etienne 2014 [30]	Case report, prospective study	One patient/two dental implants	Alveolar ridge anterior region/immediate	11	Deproteinized bovine bone mineral	Coronal part of root fragments visible through mucosal bed after removal of temporary crowns
10	Chen and Pan 2013 [31]	Case report, prospective study	One patient/one dental implant	Alveolar ridge posterior region/delayed	12	No graft	Mean buccal bone loss 0.72 mm
11	Dayakar et al., 2018 [32]	Case report, prospective study	One patient/one dental implant	Alveolar ridge anterior region/delayed	3	Bone graft	Healthy peri-implant tissue found
12	Gluckmanet al., 2017 [33]	Clinical therapy protocol	One patient/one dental implant	Alveolar ridge anterior region/immediate	12	Graft not mentioned	The SST can achieve very positive outcomes, even in the most challenging of clinical scenarios
13	Gluckman et al., 2015 [34]	Case series, retrospective study	128 patients/128 dental implants	Alveolar ridge anterior and posterior region/immediate	48	Graft not mentioned	Five dental implants failed to osseointegrate, root fragment internal exposures in 12 cases, exceeded external exposures in four cases, three root fragments developed infections, one root fragment migrated
14	Guo et al., 2018 [35]	Case report, prospective study	One patient/one dental implant	Alveolar ridge anterior region/delayed	18	No graft	Marginal bone level was stable in both vertical (13.2 mm) and horizontal (7.0 mm) directions around implant, gingiva showed no contour recession
15	Han et al., 2018 [36]	Case series, prospective clinical study	30 patients/40 dental implants	Alveolar ridge anterior and posterior region/immediate	12	No graft	Peri-implant tissues showed healthy condtion
16	Hinze et al., 2018 [37]	Case series, prospective cohort study	15 patients/17 dental implants	Alveolar ridge anterior and posterior region/immediate	3	No graft	Change in soft tissue volume in buccal contour with range-0.37 to 0.32 mm (mean-0.07 ± 0.16), gingival margin with range- 0.84 to 1.58 mm (mean 0.17 ± 0.67), Eight dental implants showed marginal gingival tissue recession
17	Petsch et al., 2017 [38]	Case report, prospective study	One patient/one dental implant	Alveolar ridge anterior region/ delayed	24	Xenograft	No clinical change in soft tissue or plaque accumulation, inter- proximal and palatal pocket probing depths where no retaining root fragment was slight increased (0.5–1 mm)
18	Schwimer et al., 2018 [39]	Technique report	One patient/2 dental implants (1 SST; 1 conventional technique)	Alveolar ridge posterior region/delayed	3–4	Xenograft	SST may help to maintain the alveolar ridge at immediate molar implant placement sites
19	Siormpas et al., 2014 [40]	Case series, retrospective study	182 patients/250 dental implants	Alveolar ridge anterior region/immediate		No graft	Five dental implants failed (two failed toosseointegrate, three had peri-implantitis), three complications due to infection of root fragment, which were treated
20	Staehler et al., 2020 [41]	Clinical therapy protocol	One patient/one dental implant	Alveolar ridge anterior region	144	No graft	The SST can provide highly asthetic and predictable outcomes
21	Sun et al., 2020 [42]	Randomized clinical study	30 patients/30 dental implants (15 SST;15 conventional flap-less approach)	Alveolar ridge anterior region	24	Deproteinized bovine bone material	Less reduction in the midfacial mucosal margins and the height of the mesial and distal papillae, as well as higher buccal plate width and height values (SST)
22	Troiano et al., 2014 [43]	Case series, prospective case study	Seven patients/10 dental implants	Alveolar ridge anterior region/delayed	6	No graft	Mean crestal bone loss 1.3 ± 0.2 mm, mesial 0.8 mm, distal 0.7 mm
23	Verma et al., 2018 [44]	Case report, prospective study	One patient/one dental implant	Alveolar ridge anterior region/immediate	12	No graft	Healthy peri-implant soft tissue and ridge were preserved
24	Zhu et al., 2018 [45]	Case series, prospective clinical study	Nine patients/10 dental implants	Alveolar ridge anterior region/immediate	12–48	No graft	Mesial bone loss 0.17 mm; distal bone loss 0.22 mm
25	Zuhr et al., 2020 [46]	Clinical case report	One patient/one dental implant	Alveolar ridge anterior region/immediate	72	No grafts	Shield around the buccal aspect of the dental implant was mobile; 8 mm buccal probing depth detected, which was surgically managed

* Abbreviation SST indicates socket-shield technique. Study has histological and clinical components and only clinical component was included in this review.

**Table 2:** Clinical studies and marginal bone resorption.

Number	Bone Modifications around Dental Implants	No. of Reported Cases and Percentage
1	Crestal bone loss of 1.3 ± 0.2 mm, mesial bone loss of 0.8 mm, distal bone loss of 0.7 mm (Troiano et al., 2014) [43]	10
2	Buccal bone loss of 0.88 mm (Baumer et al., 2013) [22]	1
3	Buccal bone loss of 0.83 ± 0.178 mm (Chen and Chen 2016) [29]	4
4	Crestal bone loss of 0.8 mm (Abadzhiev et al., 2014)	10
5	Buccal bone loss of 0.72 mm (Chen and Pan 2013)	1
6	Mesial bone loss of 0.17 mm, distal bone loss of 0.22 mm (Zhu et al., 2018) [45]	10
7	Marginal bone loss of 0.605 ± 0.06 mm (Bramanti et al., 2018) [28]	20
8	Mesial bone loss of 0.33 ± 0.43 mm, distal bone loss of 0.17 ± 0.36 mm (Baumer et al., 2017) [27]	10
9	Horizontal bone loss of 0.15 mm, vertical bone loss of 0.31 mm (Abd-Elrahman et al., 2020) [23]	20
10	Changes ranged from 0.19 mm in the midfacial region 6 mm apical to the mucosal zenith to −0.06 mm at 5 mm apical to the base of the distal papilla Mitsias et al., 2020 [4]	

**Table 3:** The quantitative description of the total number of clinical studies included in the present and complications in conjunction with the socket-shield technique.

Number of Clinical Studies	Total Number of Patients/Immediate Dental Implant Placement/Immediate Dental Implant Placement in Conjunction with the Socket-Shield Technique	Total of Dental Implant Failures Complications and Undesired Adverse Effects
25	537 patients/642 dental implants/570 dental iimplants in conjunction with the socket-shield technique	10 dental implants failed (1.75%)/123 complications and undesired adverse effects (21.58%)

**Table 4:** The frequency and percentage distribution of the dental implant failures and complications and undesired adverse effects of immediate dental implant placement in conjunction with the socket-shield technique of the included clinical studies.

Consecutive Number of the Study	Type of Complication/Author	Percentage and Number of Cases
	**Root fragment exposure**	**Total 20 (15.04%)**
**1**	Coronal part of root fragment exposed through mucosal bed (Cherel and Etienne 2014) [30]	2
**2**	Root fragment exposure (Abitbol et al., 2016) [24]	1
**3**	Root fragment internal (12 cases) and external (four cases) exposures (Gluckman et al., 2017) [33]	16
**4**	Root fragment internal (12 cases) and external (four cases) exposures (Gluckman et al., 2017) [33]	1
	**Dental implant failures**	**Total 10 (7.5%)**
**5**	Failure to osseointegrate (Siormpas et al., 2018) [40]	**2**
**6**	Due to peri-implantitis (Siormpas et al., 2018) [40]	3
**7**	Failure to osseointegrate (Gluckman et al., 2017) [33]	5
	**Root fragment with deep probing pocket depth**	**Total 2 (1.5%)**
**8**	Mesio-buccal part of 8 mm (Abitbol et al., 2016) [24]	1
**9**	Buccal probing depth of 8 mm (Zuhr et al., 2020) [46]	1
	**Root fragment infections**	**Total 6 (4.5%)**
**10**	Root fragment infections (Siormpas. et al., 2018) [40]	3
**11**	Root fragment infections (Gluckman et al., 2017) [33]	3
	**Changes in soft tissue contour**	**Total 8 (6%)**
**12**	Marginal gingival tissue recession (Hinze et al., 2018) [37]	8
	**Root fragment migration**	**Total 1 (0.75%)**
**13**	Migration of the fragment (Gluckman et al., 2017) [33]	1

## Data Availability

Data available on request from the authors.
